# A dataset of visualization methods to assessing soil profile using RES2DINV and VOXLER software

**DOI:** 10.1016/j.dib.2019.103821

**Published:** 2019-03-21

**Authors:** Safaa F. Yasir, Janmaizatulriah Jani, Mazidah Mukri

**Affiliations:** Faculty of Civil Engineering, Universiti Technology Mara, UiTM, Shah Alam, 40450, Malaysia

**Keywords:** Geophysics technique, Visualization software, Soil profile, Site investigation

## Abstract

This data illustration the similarity and accuracy of two subsurface profile analysis software which is RES2DINV and VOXLER. Electrical resistivity imaging methods was conducted as a geophysical technique to get subsurface profile were borehole had previously been made in the same locations. The General Department of Geoscience (JMG) conducted the drilling of the borehole in three locations which is Kampung Bangkahulu, Gemas, Kampung Semerbok, Rembau and Felda Bukit Rokan Utara. The 2D resistivity image from RES2DINV and the 3D image from VOXLER was highly matching the subsurface profile compared with borehole data log. The depth of the resistivity was 76.8, 87.2 and 39.4 respectively for the sites. This two software gave more clearly interpreted result for investigate the sub ground and geological formations.

**Table:** Specifications table

Subject area	*Geo-Physics, Hydrology*				
More specific subject area	*Electrical Resistivity Imaging technique*				
Type of data	*text file*				
How data was acquired	*ABEM SAS 4000 equipment produce Electrical Resistivity Imaging Raw data*				
Data format	*Raw, analyzed*				
Experimental factors	*Raw data analyzed by RES2DINV & VOXLER software.*				
Experimental features	*Schlumberger configurations was used with (81) and (41) numbers of electrode and resistivity land with 100 m length. The electrode was connected to resistivity land cables using jumper cable.*				
Data source location	Site	First electrode		Last electrode	
		N	E	N	E
	Site 1	02° 35′ 58.1″	102° 34′ 14.0″	02° 35′ 56.1″	102° 34′ 26.6″
	Site 2	02° 28′ 13.5″	102° 08′ 06.6″	02° 28′ 23.2″	102° 07′ 59.1″
	Site 3	02° 39′ 43.0″	102° 25′ 14.3″	02° 39′ 37.5″	102° 25′ 12.9″
Data accessibility	*Data is with this article*				
Related research article	*Yasir, S.F., J. Jani, and M. Mukri, Geophysical measurement for estimation of groundwater hydraulic properties. Data in brief, 2018. 21: p. 907–910.*

**Table:** 

**Value of the data** 1∗ RES2DINV software can explicate the geological formations based on its resistivity while VOXLER software can interpreted specific formation by determine its resistivity. 2∗ Isosurface option in Voxler Software can mitigate the ambiguity of overlap in resistivity of geological formations. 3∗ This data showed how this two software can work together for good subsurface interpretation.

## 1 Data

The electrical resistivity imaging contains many electrodes which are pinned into the ground (Fig. 2) and a computer-based system scans the whole array, thus realizing a combined sounding and profiling. For 2D imaging, the pseudo section of apparent resistivity will acquisition by the effective investigates is a series of depth range on a profile line. Electrical resistivity imaging encompasses a measuring a series of constant separation traversing and with each successive traverse, the electrode separation being increased. The apparent resistivity will be plot as a contoured section based on information from greater depth which come from increasing separation. Thus, the spatial variation in resistivity in the vertical cross-section will qualitatively reflect as can see in Table 1, Table 2. The electrode spacing will determine the depth of penetration and Length of profile.

**Fig. 1:**
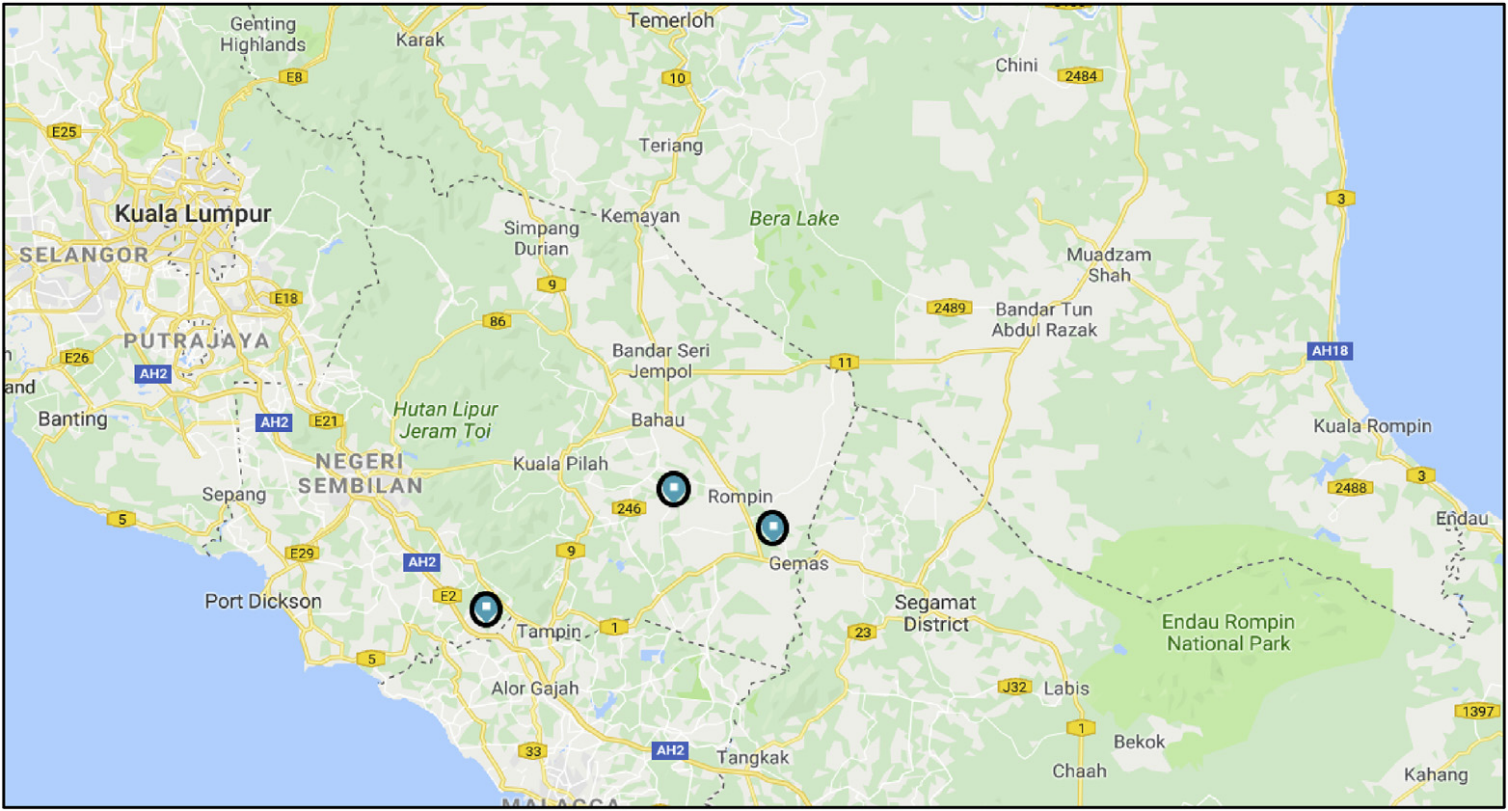
Locations of the resistivity test (Google Map).

**Fig. 2:**
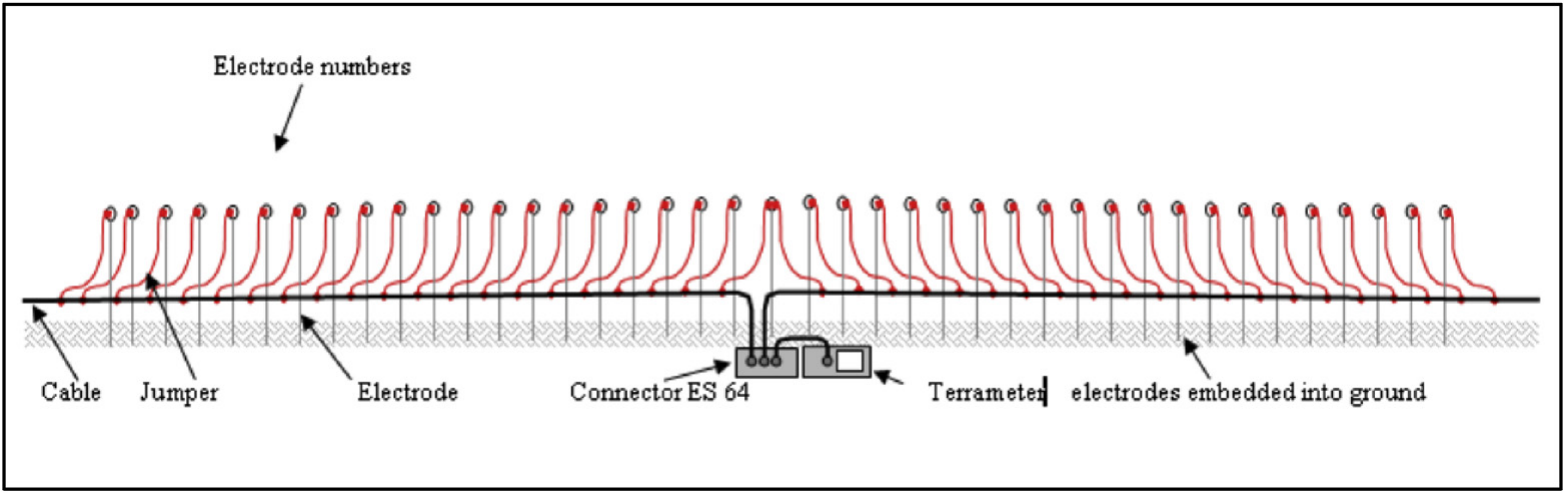
Schematic diagram of electrical resistivity set up for field measurement.

**Table 1:** Resistivity of various rocks and minerals [2].

Material	Resistivity (Ω-m)
**Igneous/Metamorphic**	
Granite	5 × 10^3^–10^8^
Weathered granite	1–10^2^
Basalt	10^3^–10^6^
Quartz	10^3^–2 × 10^6^
Marble	10^2^ –2.5 × 10^8^
Schist	20–10^4^
**Sediments**	
Sandstone	8–4 × 10^3^
Conglomerate	2 × 10^3^–10^4^
Shale	20–2 × 10^3^
Limestone	50–4 × 10^2^
**Unconsolidated sediment**	
Clay (wet)	20
Marl	1–70
Clay	1–100
Alluvium	10–800
**Groundwater**	
Salt water	0.2
Fresh water	10–100

**Table 2:** Resistivity of some rock, minerals and water types [3].

Material	Resistivity (Ohm-m)
Groundwater (in igneous rock)	30–150
Surface water (in igneous rock)	30–500
Sea water	0.2
Sandstone	33–6700
Sand and Gravel	100–180
Schist	10–1000
Top Soil	67–100
Clayey Soil	100–133
Clay	1–67
Limestone	67–1000
Sandy Soil	670–1330
Saline water 3%	0.15
Saline water 20%	0.05

Voxler, a 3D scientific visualization program, oriented originally toward three-dimensional data display and volumetric performance. Voxler can utilize 2D grids including DEM files, images, and scattered point data also emphasis is on 3D volumes. Voxler can display streamlines, contour maps, vector plots, isosurfaces, 3D scatter plots, image slices, direct volume recall, three-dimensional block models, well traces, and more [1] Computational modules include three-dimensional gridding, resampling, numerous lattice operations, and image transformations. Voxler is designed for displaying XYZC data, where C is a component variable at each X, Y, and Z location. The resistivity values of several formation are also given in Table 1, Table 2.

## 2 Experimental design, materials, and methods

A 2-D electrical resistivity imaging method was employed in this work with a multi electrode resistivity meter system. For the site Kampung Bangkahulu Gemas and Kampung Semerbok, Rembau, Schlumberger configurations was used with (81) numbers of electrode and (4) resistivity land cables each one is 100 m [4]. The third location (Felda Bukit Rokan Utara), (41) numbers of electrode was used and (2) resistivity land cables each one is 100 m with same configurations (Schlumberger). The electrode was coupled to resistivity land cables via jumper cable. The whole line of electrical resistivity investigation line is 400 m for the first and second site and 200 m for the third site where 5-m spacing. Fig. 1 show the locations of the resistivity test were conducted and the coordinates of the resistivity survey line are showing in Table 3. Electrode number 1 (E1) and electrode number 81 (E 81 for the first and second site) and Electrode number 41 (E41 for the third site) must be defined. As the connections of lines completed and Terrameter were set up, all data was automatically recorded by the Terrameter SAS 4000 and LUND Imaging System and the data was saved in the Terrameter main unit before transferring to computer. The data collected in the survey is then converted to image using RES2DINV software. The schematic layout of the electrode arrangement and connections is displayed in Fig. 2.

**Table 3:** Coordinate of the resistivity survey line.

Site	First electrode		Last electrode	
	N	E	N	E
Site 1	02° 35′ 58.1″	102° 34′ 14.0″	02° 35′ 56.1″	102° 34′ 26.6″
Site 2	02° 28′ 13.5″	102° 08′ 06.6″	02° 28′ 23.2″	102° 07′ 59.1″
Site 3	02° 39′ 43.0″	102° 25′ 14.3″	02° 39′ 37.5″	102° 25′ 12.9″

**Fig. 3:**
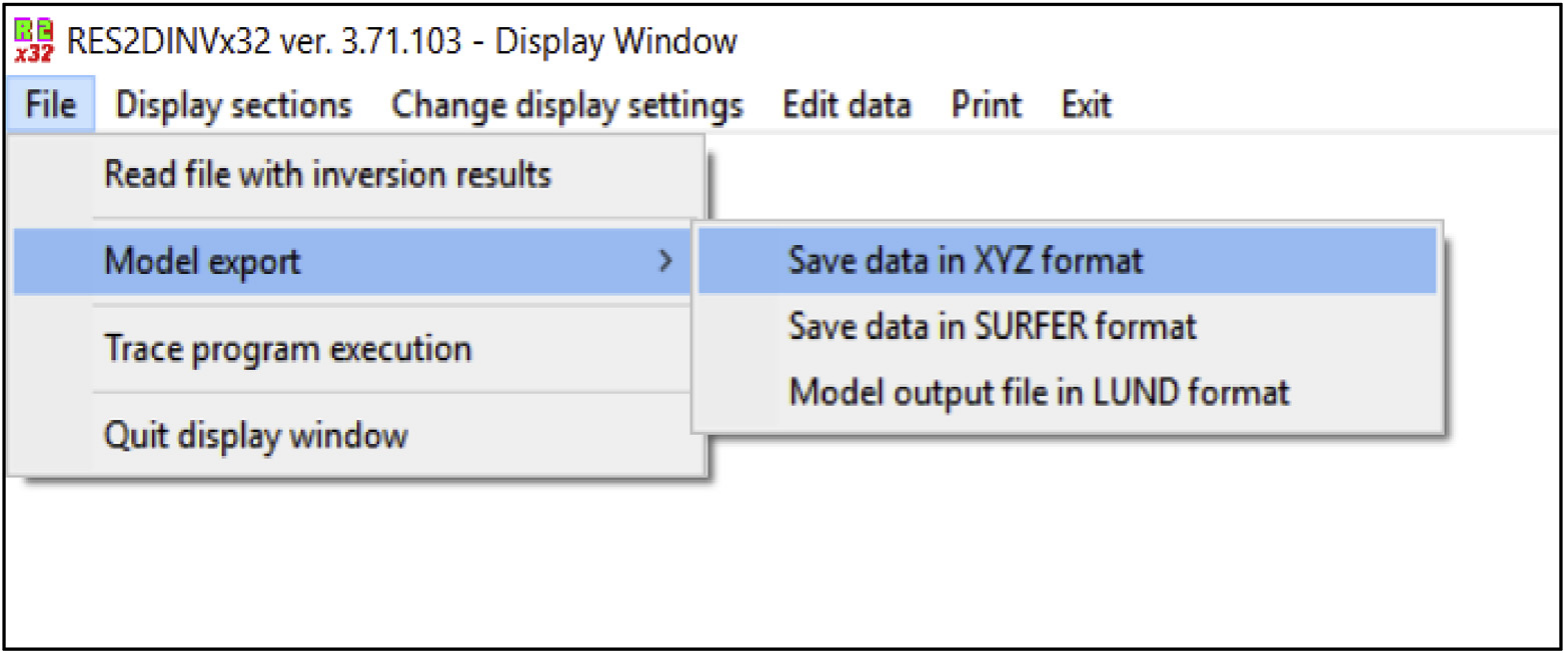
Save data in XYZ format.

By RES2DINV Software, the raw data of electric resistivity was converted to (XYZ) file and saved as showing in Fig. 3. The (XYZ) format used as input data for Voxler software to generate 3D visualize picture. The General Department of Geoscience (JMG) conducted the borehole drilling in Kampung Bangkahulu, Gemas as shown in Appendix A. The truck mounted rig drilling machine was used and conduct with down the hole hammer method. The drilling depth was until 100 m and the drilling show three layers. The first layer was reddish clay with thickness 3 m and second layer was 6 m whitenish clay. The third layer was schist with 91 m thickness. The General Department of Geoscience (JMG) also conducted the borehole drilling in Kampung Semerbok, Rembau and Felda Bukit Rokan Utara (Appendix A). The data log shows two layers only for second location (Kampung Semerbok, Rembau) which is reddish clay and schist, while it shows three layers which is silty sand, shale and schist for the third site. To accomplish the objectives of this study, 2D electrical resistivity imaging from RES2DINV and 3D resistivity image from VOXLER software compared with data from borehole data log. Appendix B show the 2D and 3D images for subsoil for the three sites.

## 3 Limitations

1. There is overlap in resistivity of geological formation especially with the aquifer in RES2DINV software which can distinguished by isosurface option in Voxler Software.
2. Since the data in this paper conducted by 2D electrical resistivity imaging, the Y direction was assumed zero when using Voxler Software.
3. There are no limitations from aspect of depth were the raw data collected from field test of electrical resistivity imaging can directly use by Voxler Software after convert the (DAT) format file to (EXCEL SHEET) format.
