# Supplementary material for: A dataset of visualization methods to assessing soil profile using RES2DINV and VOXLER software
Source: Data Brief. 2019 Mar 21;24:103821. doi: 10.1016/j.dib.2019.103821 (PMC6441720; doi:10.1016/j.dib.2019.103821)
Supplement: Multimedia component 1 [file mmc1.docx]

Conflict of Interest Form

We wish to confirm that there are no known conflicts of interest associated with this

publication and there has been no significant financial support for this work that could have

influenced its outcome.

We confirm that the manuscript has been read and approved by all named authors and that

there are no other persons who satisfied the criteria for authorship but are not listed. We

further We confirm that the order of authors listed in the manuscript has been approved by all of

us.
