# Supplementary material for: A dataset of visualization methods to assessing soil profile using RES2DINV and VOXLER software
Source: Data Brief. 2019 Mar 21;24:103821. doi: 10.1016/j.dib.2019.103821 (PMC6441720; doi:10.1016/j.dib.2019.103821)
Supplement: Multimedia component 2 [file mmc2.docx]

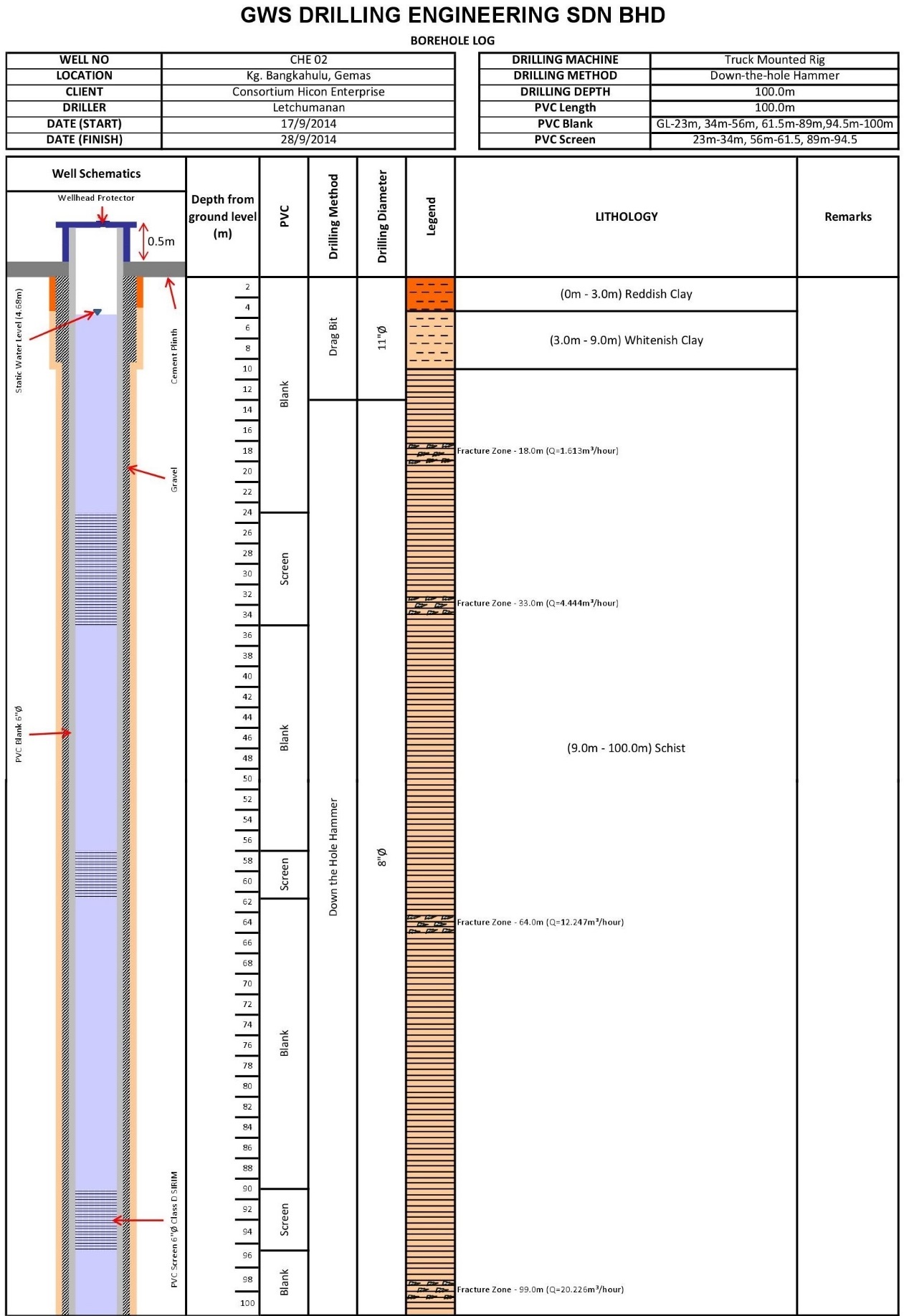


Borehole Log of Kg. Bangkahulu, Gemas.


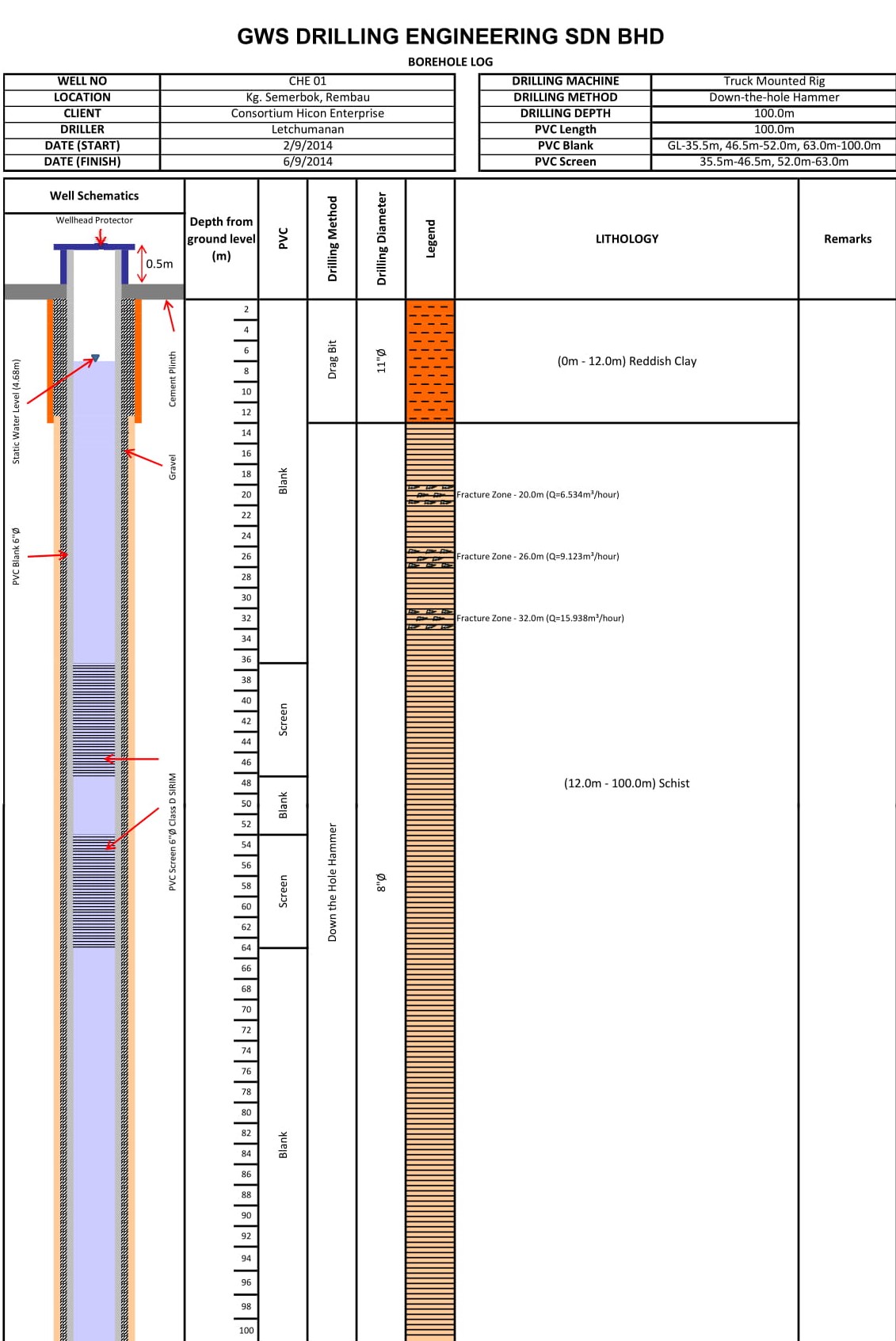


Borehole Log of Kampung Semerbok, Rembau.


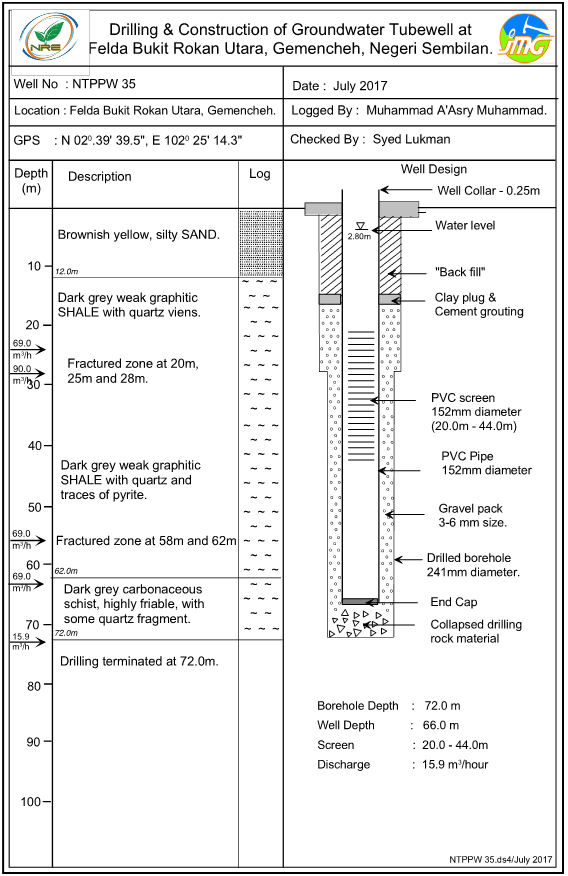


Borehole Log of Felda Bukit Rokan Utara.
