# Supplementary material for: A dataset of visualization methods to assessing soil profile using RES2DINV and VOXLER software
Source: Data Brief. 2019 Mar 21;24:103821. doi: 10.1016/j.dib.2019.103821 (PMC6441720; doi:10.1016/j.dib.2019.103821)
Supplement: Multimedia component 3 [file mmc3.docx]

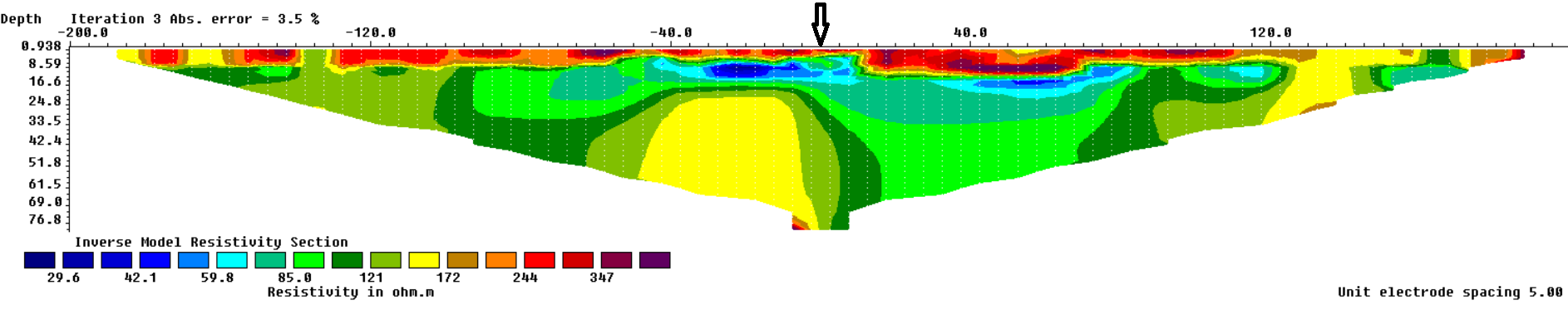


Resistivity image of Kampung Bangkahulu, Gemas site.


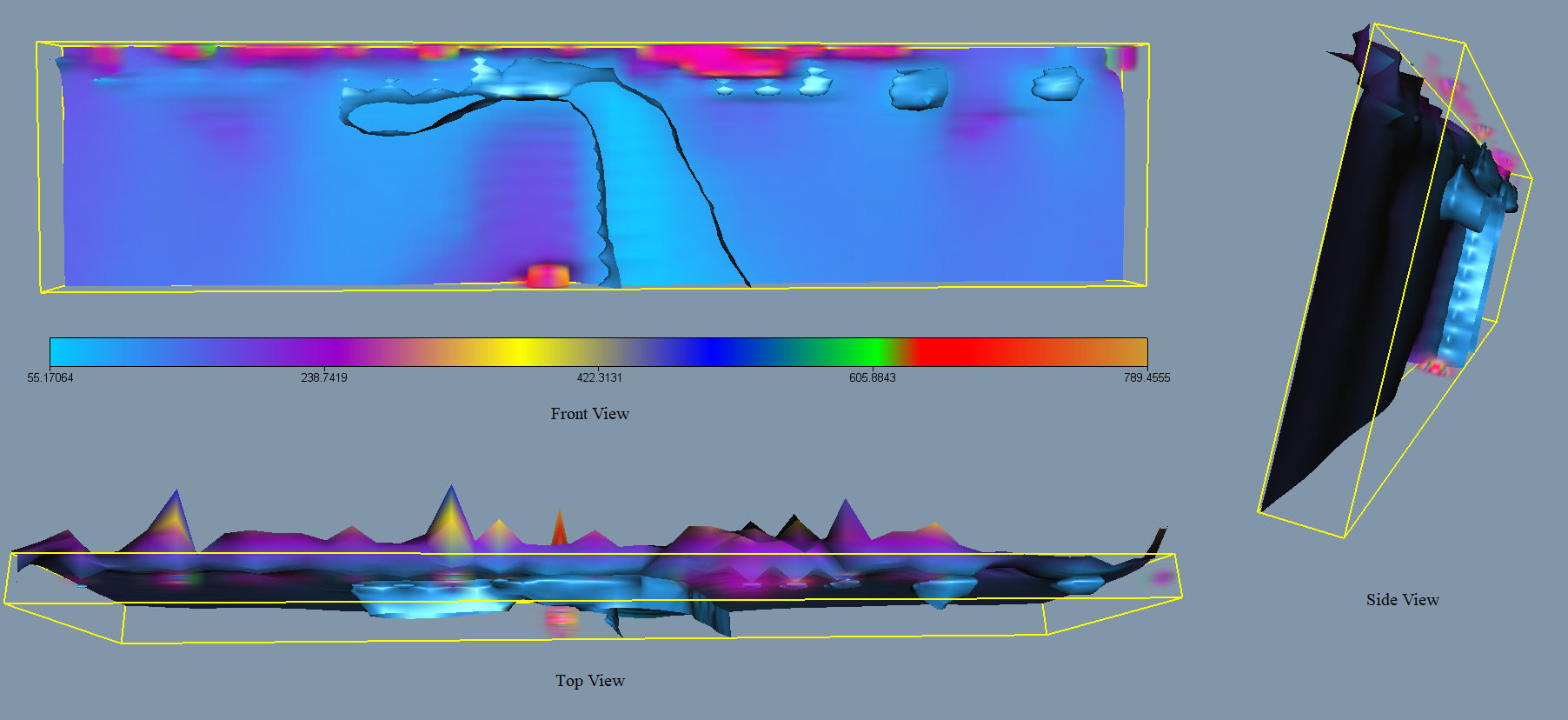


Different 3D views of Kampung Bangkahulu, Gemas site.


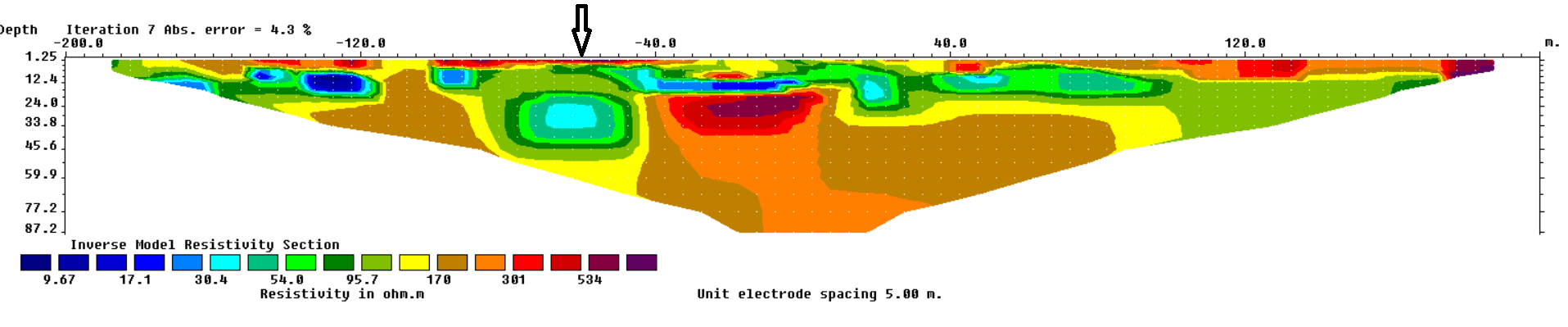


Resistivity image of Kampung Semerbok, Rembau site.


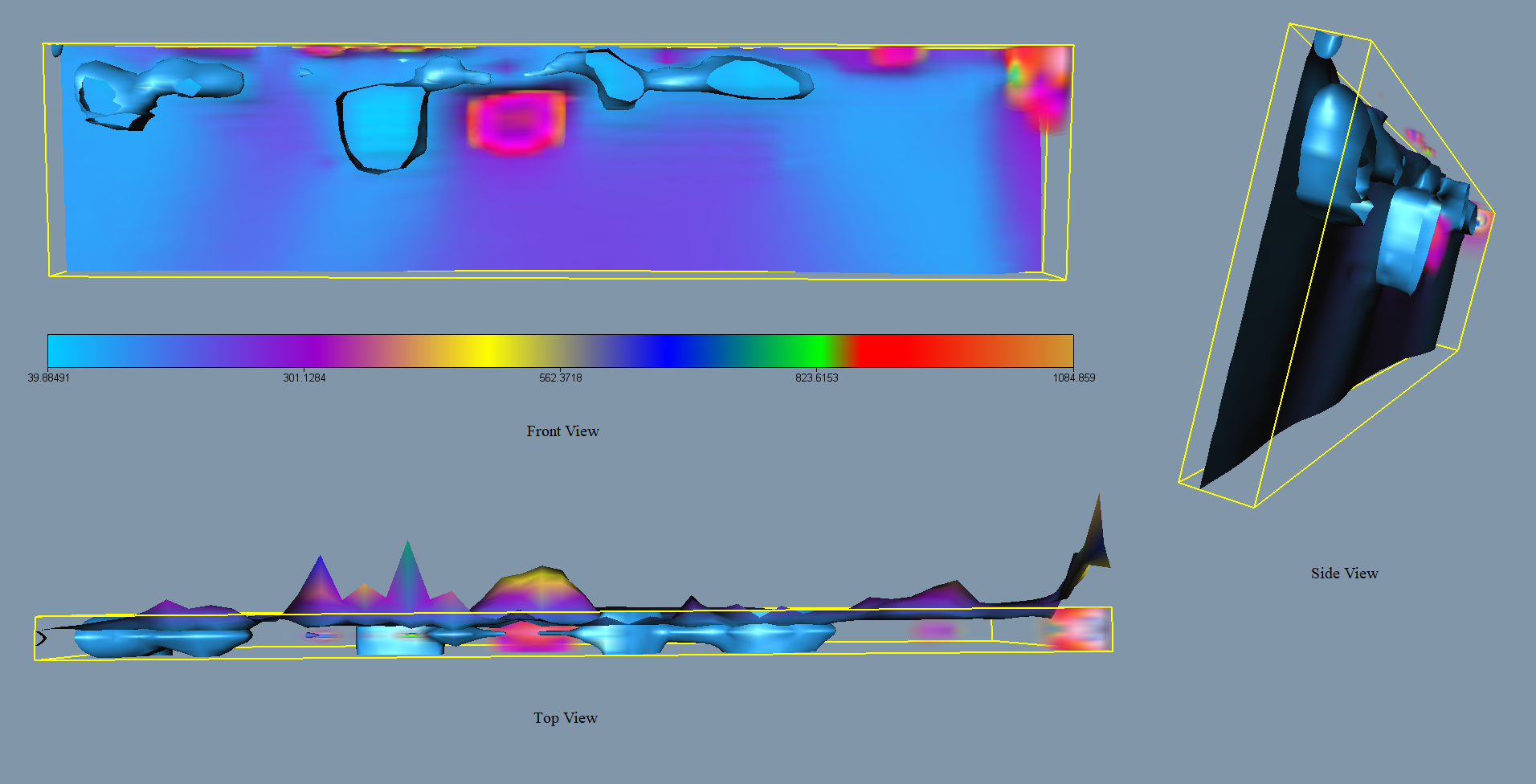


Different 3D views of Kampung Semerbok, Rembau site.


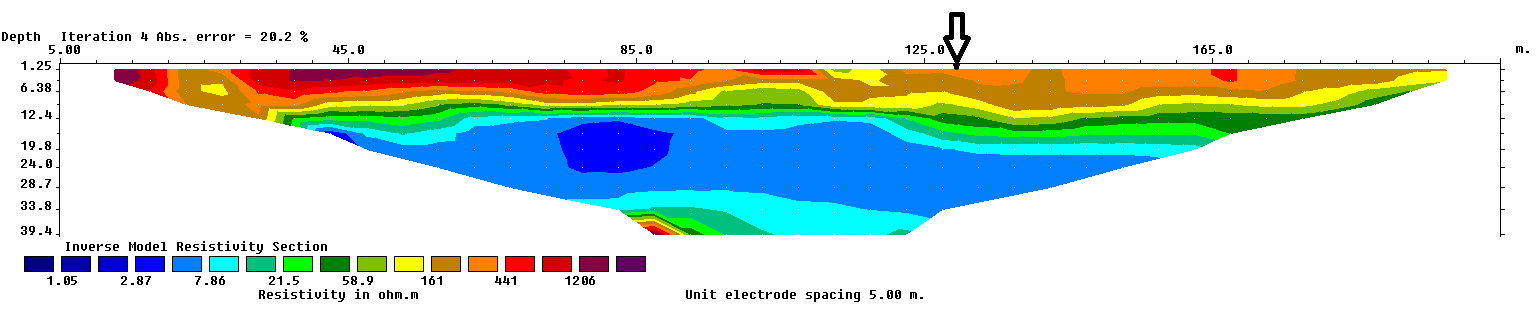


Resistivity image of Felda Bukit Rokan Utara site.


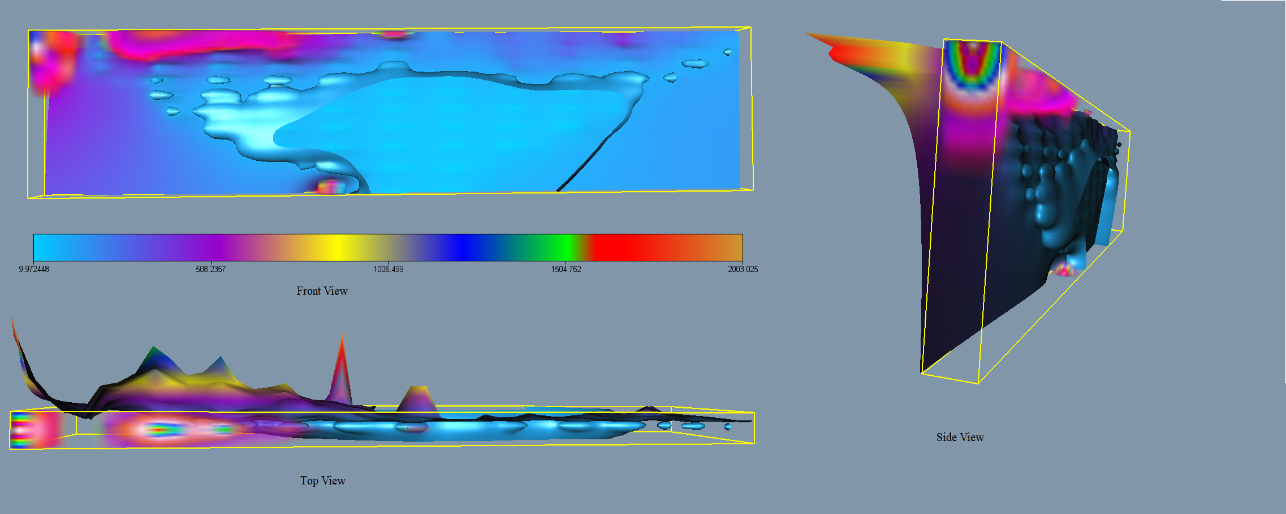


Different 3D views of Felda Bukit Rokan Utara site.
